# Enhanced U-Net-Based Deep Learning Model for Automated Segmentation of Organoid Images

**DOI:** 10.3390/bioengineering12111216

**Published:** 2025-11-07

**Authors:** Maath Alani, Hamid A. Jalab, Selin Pars, Bahaa Al-mhanawi, Rowaida Z. Taha, Ernst J. Wolvetang, Mohammed R. Shaker

**Affiliations:** 1 Australian Institute for Bioengineering and Nanotechnology, The University of Queensland, Brisbane, QLD 4072, Australia; s.pars@uq.edu.au (S.P.); b.almhanawi@uq.edu.au (B.A.-m.); 2 Information and Communication Technology Research Group, Scientific Research Centre, Alayen University, Nasiriyah 64001, Thi Qar, Iraq; hamid.a@alayen.edu.iq; 3 Neurological Disorders Research Center, Qatar Biomedical Research Institute, Hamad Bin Khalifa University, Qatar Foundation, Education City P.O. Box 34110, Doha, Qatar; rotaha@hbku.edu.qa; 4 College of Health and Life Sciences, Hamad Bin Khalifa University, Qatar Foundation, Education City P.O. Box 34110, Doha, Qatar

**Keywords:** deep learning, organoids, U-net, image segmentation, dice similarity coefficient, Jaccard index, convolutional neural network

## Abstract

Organoids have emerged as powerful in vitro models for studying human development, disease mechanisms, and drug responses. A critical aspect of organoid characterisation is monitoring changes in size and morphology during culture; however, extracting these metrics from high-throughput imaging datasets is time-consuming and often inconsistent. Automated deep-learning approaches can overcome this bottleneck by providing accurate and reproducible image analysis. Here, we present an enhanced U-net-based segmentation model that incorporates region-of-interest refinement to improve the delineation of organoid boundaries. The method was validated on bright-field organoid images and demonstrated robust performance, achieving an accuracy of 98.15%, a dice similarity coefficient of 97.19%, and a Jaccard index of 94.53%. Compared with conventional segmentation methods, our model provides superior boundary detection and morphological quantification. These results highlight the potential of this approach as a reliable tool for high-throughput organoid analysis, supporting applications in disease modelling, drug screening, and personalised medicine.

## 1. Introduction

Organoids, three-dimensional structures derived from stem cells, have emerged as powerful models for studying human development [1], disease mechanisms [2], and drug responses [3,4]. These miniature, organ-like systems recapitulate key aspects of human physiology and pathology [5], providing new opportunities to investigate complex biological processes in vitro. Despite their potential, the field faces significant challenges in reproducibility, scalability, and phenotypic characterisation [6,7]. High variability in organoid growth and morphology often leads to inconsistent results, making it difficult to derive reliable biological insights [8].

Artificial intelligence (AI) approaches, particularly deep learning, are increasingly being applied to overcome such limitations. Advanced segmentation models can precisely delineate organoid boundaries, enabling accurate quantification of size, shape, and structural features. Standardising image analysis through AI not only reduces variability but also improves reproducibility across laboratories [9]. When combined with high-throughput imaging, deep learning provides a scalable solution for analysing large organoid datasets, accelerating the identification of disease signatures and therapeutic targets.

Quantitative image analysis has long been central to medical research, including applications such as pathological grading and cancer diagnostics [10,11,12]. At the core of these efforts lies image segmentation, a process that remains technically challenging due to complex tissue structures and variable image quality. Traditional techniques, such as histogram-based thresholding, contour detection, and watershed algorithms [13], frequently struggle with noisy backgrounds and complex morphologies. While supervised learning approaches introduced in the 1990s improved performance [14], deep learning, particularly convolutional neural networks (CNNs) [15], has since transformed the field.

Semantic segmentation using CNNs has evolved rapidly, moving from patch-based approaches to architectures that exploit full-image context. U-net [16,17] and V-net [17] have become cornerstones in biomedical image segmentation, leveraging encoder–decoder structures to capture both local and global features. Enhancements such as ResNet feature extractors [18], multi-scale input layers [19], and scalable frameworks like Mask R-CNN [20] have further advanced segmentation accuracy and applicability across domains.

Compared with classical methods, CNN-based approaches consistently achieve superior performance and are now widely adopted in medical and biological imaging tasks, including histological analysis and vascular segmentation [19]. Building on these advances, we present an enhanced U-net-based algorithm for organoid segmentation. Our method incorporates a region-of-interest (ROI) refinement strategy to address challenges such as blurred boundaries and irregular morphologies, improving segmentation accuracy and consistency. We further demonstrate that this fully automated pipeline can reduce the workload associated with organoid image analysis and provide unbiased quantitative metrics.

## 2. Materials and Methods

### 2.1. Human Pluripotent Stem Cell Culture

Human pluripotent stem cell (hPSC) lines, including the GENEA22 and H9 human embryonic stem cell line as well as three human-induced pluripotent stem cell lines (1 control and 3 epilepsy lines) [21,22], were used for brain organoid generation. Cells were maintained on Geltrex™ LDEV-Free Reduced Growth Factor Basement Membrane Matrix (Thermo Fisher Scientific, Waltham, MA, USA, Cat. #A1413202) at a 1:100 dilution and cultured in mTeSR™ Plus medium (Stem Cell Technologies, Vancouver, BC, Canada, Cat. #100-0276), with daily medium changes. Colonies were passaged at 70–80% confluency using 0.5 mM EDTA (Thermo Fisher Scientific, MA, USA, Cat. #15575020) followed by Accutase Enzyme Cell Detachment Medium (Thermo Fisher Scientific, MA, USA, Cat. #00-4555-56). Cells were incubated under normoxic conditions (37 °C, 5% CO_2_, humidified atmosphere).

### 2.2. Brain Organoid Generation

Cortical organoids were generated from hPSCs using a previously described protocol with slight modifications [23]. The hPSC lines [6,7] were included as sources for organoid derivation. Stem cells were expanded as colonies on a thin Geltrex layer and maintained in mTeSR Plus medium. Colonies were passaged every 4–7 days using 0.5 mM EDTA and singularised with Accutase at 65–75% confluency.

For embryoid body formation, 2.46 × 10^6^ hPSCs were seeded per well in an AggreWell800 plate (Stem Cell Technologies, Vancouver, BC, Canada, Cat. #34815) with 3 mL of mTeSR medium containing 10 µM of ROCK inhibitor (Sapphire Bioscience, Redfern, Australia, Cat. #10005583) for 24 h (Day 1). Embryoid bodies were collected, washed with DMEM:F12 medium (Thermo Fisher Scientific, MA, USA, Cat. #11320082), and transferred to Stage 1 medium [DMEM:F12 supplemented with 2% B27 without vitamin A (Thermo Fisher Scientific, MA, USA, Cat. #12587001), 1% N2 (Thermo Fisher Scientific, MA, USA, Cat. #17502048), 1% non-essential amino acids (Thermo Fisher Scientific, MA, USA, Cat. #11140050), 1% Penicillin–Streptomycin (Thermo Fisher Scientific, MA, USA, Cat. #15070063), and 55 µM of β-mercaptoethanol (Thermo Fisher Scientific, MA, USA, Cat. #21985023)], containing 2.5 µM of dorsomorphin (Sigma Aldrich, Burlington, MA, USA, Cat. #P5499) and 10 µM of SB-431542 (Miltenyi Biotec, Bergisch Gladbach, Germany, Cat. #130-106-543). One well of AggreWell800-derived embryoid bodies was plated into two 10 cm dishes and maintained until Day 6 with daily medium changes.

On Day 6, embryoid bodies were transferred to ultra-low-attachment 96-well plates to prevent fusion and cultured in Stage 2 medium [Neurobasal-A (Thermo Fisher Scientific, MA, USA, Cat. #10888022) with 2% B27 without vitamin A, 1% GlutaMAX (Thermo Fisher Scientific, MA, USA, Cat. #35050061), 1% Penicillin–Streptomycin], supplemented with 20 ng/mL of EGF (Stem Cell Technologies, BC, Canada, Cat. #78006.1) and 20 ng/mL of FGF-2 (Stem Cell Technologies, BC, Canada, Cat. #78003). Medium was changed daily from Day 6 to Day 15 and every other day until Day 22. From Day 22 onward, organoids were cultured in Stage 2 medium supplemented with 20 ng/mL of BDNF (Stem Cell Technologies, BC, Canada, Cat. #78005.1), 20 ng/mL of NT-3 (Stem Cell Technologies, BC, Canada, Cat. #78074.1), 200 µM of L-ascorbic acid (Sigma Aldrich, MA, USA, Cat. #A4544), 50 µM of cAMP (Stem Cell Technologies, BC, Canada, Cat. #73886), and 10 µM of DHA (Stem Cell Technologies, BC, Canada, Cat. #D2534), with feeding every second day.

At Day 46, organoids were transferred to ultra-low-attachment 24-well plates and maintained in Stage 4 medium [Neurobasal-A with 2% B27 Plus (Thermo Fisher Scientific, MA, USA, Cat. #A3582801), 1% GlutaMAX, 1% Penicillin–Streptomycin], refreshed every 4–5 days.

### 2.3. Data Acquisition

**Figure 1:**
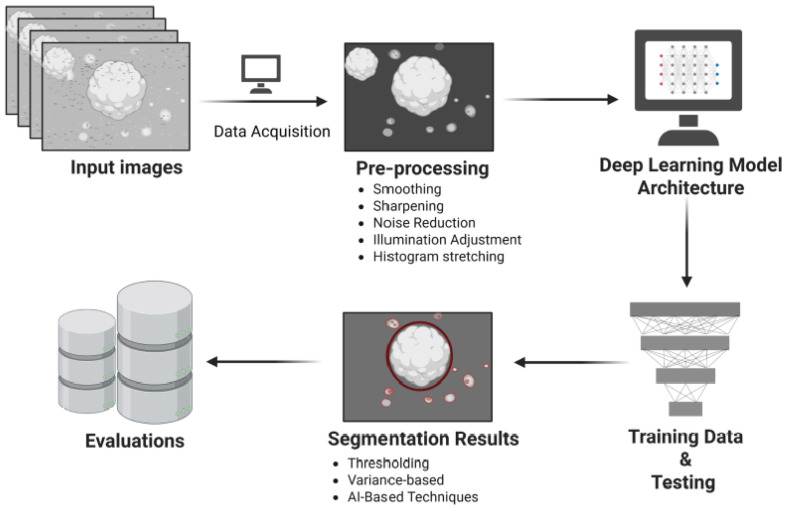
Workflow of the proposed organoid segmentation pipeline. The schematic illustrates the major steps of the enhanced U-net-based segmentation framework. Input bright-field images are first acquired (Data Acquisition) and subjected to preprocessing (smoothing, sharpening, noise reduction, illumination adjustment, and histogram stretching) to improve image quality. The processed images are then used for training and testing within the deep-learning model architecture. In the deep learning model architecture, red and blue nodes represent the input and output layers, respectively, while grey nodes denote hidden layers. Segmentation outputs are generated using thresholding, variance-based, and AI-driven techniques, with red outlines indicating the detected organoid boundaries in the segmentation results. Finally, performance metrics are evaluated to assess segmentation accuracy.

Individual organoids were labelled by well position and imaged longitudinally from Day 6 onwards. Bright-field images of control and epilepsy-derived organoids were acquired using an Olympus CKX53 inverted microscope with a 4× objective, 10× ocular, and a 5-megapixel Olympus SC50 colour camera. Images were captured with Olympus cellSens software (v2.3) and saved in TIFF format. The proposed system consists of two main steps: the first step is the determination of the boundaries of the cell, and the second step is ROI segmentation from the organoid images using the proposed segmentation method. The organoid images vary from one captured image to another. This variation includes the shape and size of the cell. In this study, the steps of the proposed method are as shown in Figure 1.

### 2.4. Deep-Learning Model Architecture

**Figure 2:**
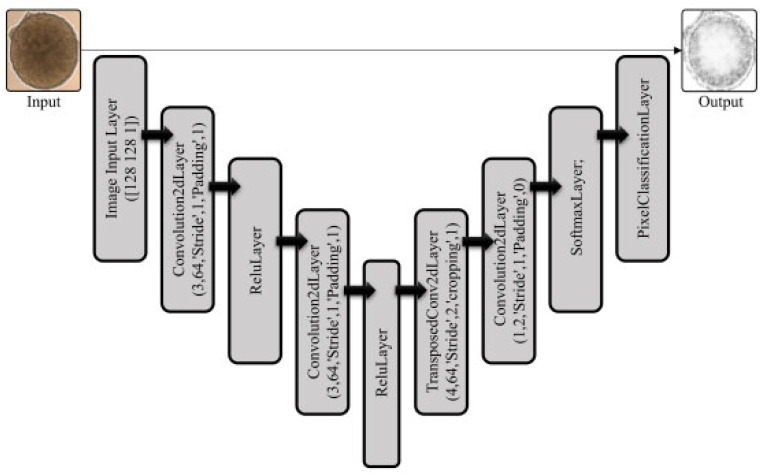
Architecture of the CNN-based segmentation model. Schematic block diagram showing the overall structure of the model, including the input image layer, stacked convolutional layers with ReLU activations, pooling and transposed convolutional layers for feature mapping, and final Softmax and pixel classification layers producing the segmented organoid output.

A convolutional neural network (CNN) segmentation model was implemented, comprising multiple convolutional layers, ReLU activation [24,25], pooling layers, and a pixel classification layer (Figure 2). Convolutional kernels were applied with fixed stride values to balance feature extraction and computational efficiency. Max-pooling layers reduced dimensionality, while a Softmax activation function assigned probabilities to each class. The pixel classification layer performed semantic segmentation by assigning categorical labels to each pixel, excluding undefined labels during training for improved accuracy.

The model was trained using the crossentropyex loss function to minimise the difference between predicted and true pixel values. Hyperparameters, including learning rate, batch size, and kernel size, were tuned to optimise convergence. Data augmentation strategies (rotation, flipping, scaling) were applied to improve robustness. Model performance was assessed on 128 × 128 pixel test images representing diverse organoid morphologies, with segmentation accuracy measured against ground-truth masks [26,27,28].

### 2.5. Image Preprocessing

**Figure 3:**
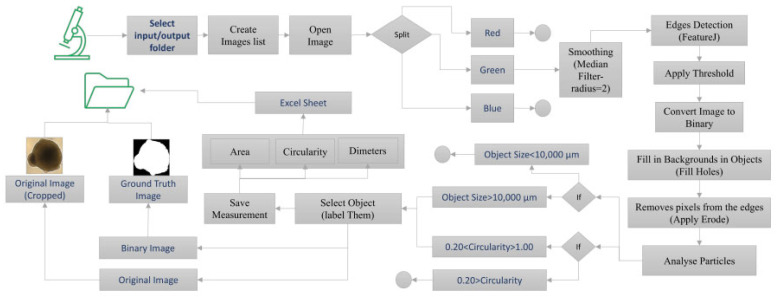
Image preprocessing workflow for organoid bright-field datasets. Flowchart depicting the preprocessing sequence applied to raw bright-field images, including channel separation, median filtering, edge detection, thresholding, binary conversion, hole filling, erosion, and particle analysis for measurement of area, diameter, and circularity.

To prepare a high-quality dataset, bright-field organoid images (2560 × 1920 pixels) were processed using ImageJ 1.54p macros (Figure 3). Files were organised into input/output folders and split into red, green, and blue channels, with the green channel selected for further analysis. Median filtering was applied to smooth edges, followed by FeatureJ-based edge detection. Images were thresholded, converted to binary, and processed with “Fill Holes” and “Erode” functions to remove background noise. Particle analysis was performed to extract area, circularity, and diameter measurements, and objects were labelled. Binary and original images were then cropped to generate paired ground-truth masks and training inputs.

### 2.6. Training of CNN Segmentation Model

The CNN was configured with four convolutional layers to reduce model complexity and avoid overfitting. A stride of 2 was applied uniformly across layers, equivalent to pooling stride, reducing computational burden while preserving feature resolution. The network was trained using the Adam optimiser with an initial learning rate of 0.001 for 300 epochs and categorical cross-entropy loss. Training and validation curves of accuracy and loss were used to determine optimal parameters (Figure 3).

## 3. Results and Discussion

The proposed segmentation framework was designed in two main steps: (i) identification of organoid boundaries and (ii) ROI segmentation. This strategy was necessary to accommodate the variability in organoid morphology across captured bright-field images. The workflow of the method is summarised in Figure 1. Training and testing were performed on an Intel i7-6700HQ CPU (2.6 GHz) with 8 GB RAM and a NVIDIA GTX 950 GPU, running MATLAB 2019b. Input images were resized to 240 × 240 pixels for training (Figure 2), which reduced computational complexity and accelerated processing while preserving structural information. Preprocessing steps are illustrated in Figure 3. Before training, the dataset of 1000 bright-field organoid images were divided into the following: 70% for training; 10% for validation, and 20% for testing, which is a final evaluation after training.

### 3.1. Evaluation Metrics

To assess the performance of the segmentation model, three widely used quantitative metrics were employed:

1. Accuracy: the ratio of correctly classified pixels to the total number of pixels,
  $$Accuracy=\frac{TP+TN}{TP+FP+TN+FN}$$
2. Dice Similarity Coefficient: a measure of spatial overlap between prediction and ground truth,
  $$Dice=\frac{2TP}{2TP+FP+FN}$$
3. Jaccard Index: the proportion of correctly predicted pixels relative to the union of prediction and ground truth,
  $$Jaccard=\frac{TP}{TP+FP+FN}$$

where TP, TN, FP, and FN represent true positive, true negative, false positive, and false negative pixels, respectively.

### 3.2. Model Testing

**Figure 4:**
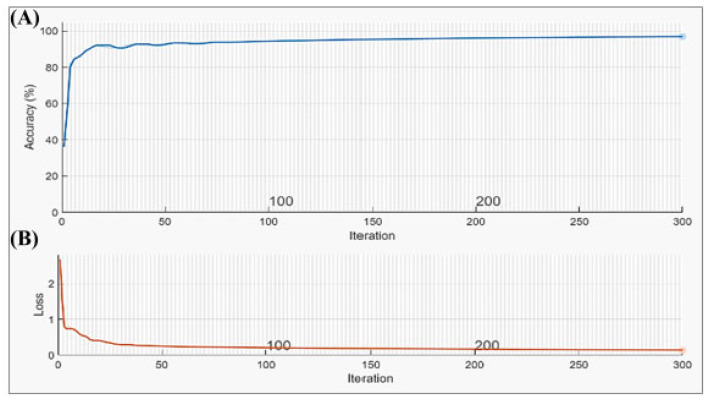
Training performance of the CNN model. (**A**) Accuracy curve showing rapid improvement within the first ~30 iterations and stabilisation at >90% across 300 iterations. (**B**) Loss curve demonstrating a sharp decline during early iterations and convergence to near-zero values as training progressed.

**Figure 5:**
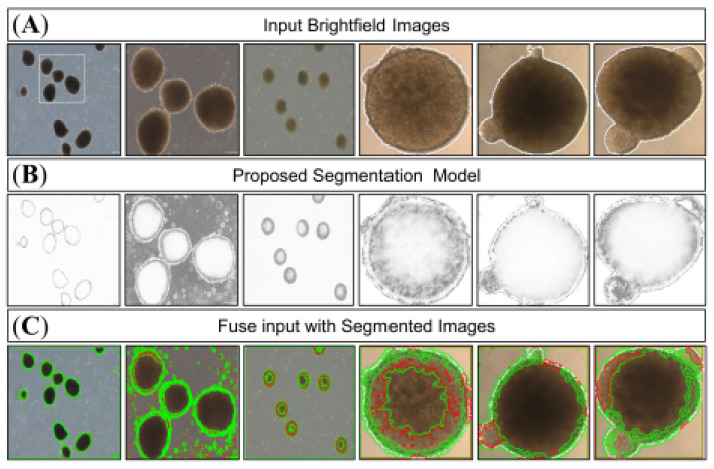
Segmentation of bright-field organoid images using the proposed CNN model. (**A**) Representative input bright-field images of organoids. (**B**) Predicted segmentation masks generated by the CNN-based model, highlighting organoid boundaries. (**C**) Fused overlays of the original bright-field images with the segmentation outputs (green outlines), illustrating accurate detection and delineation of organoid structures across varying morphologies and sizes.

**Figure 6:**
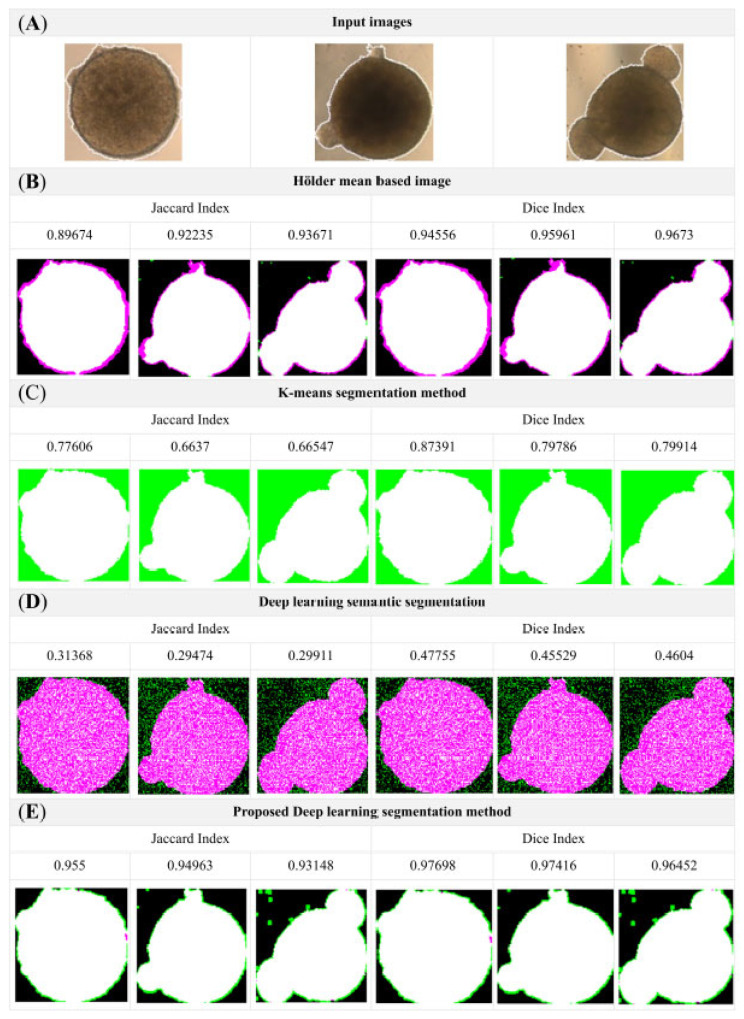
Comparison of segmentation performance across conventional and deep-learning-based methods. (**A**) Representative input bright-field images of organoids. (**B**) Hölder mean-based segmentation results, with high accuracy reflected by Jaccard and Dice indices ranging from 0.8967 to 0.9673. (**C**) K-means clustering segmentation, yielding moderate performance with indices between 0.6637 and 0.8739. (**D**) Standard deep-learning semantic segmentation, which performed poorly with Jaccard values of 0.29–0.48 and Dice indices of 0.31–0.46. (**E**) Proposed enhanced U-net-based model, showing superior segmentation with consistently high Jaccard (0.93–0.95) and Dice indices (0.96–0.97). Quantitative metrics are shown above each segmentation panel for direct comparison of methods.

The trained model was evaluated on a test set of 128 × 128 pixel organoid images (Figure 4). Representative segmentation results are shown in Figure 5. Qualitative inspection demonstrated that the proposed method provided superior delineation of organoid structures compared with baseline approaches, including Hölder mean-based segmentation, K-means clustering, and standard semantic deep-learning segmentation (Figure 6). For computational efficiency, the proposed segmentation model achieved an average inference time of 0.0486 ± 0.0045 s per image (128 × 128 resolution) on an NVIDIA GTX 950 GPU, corresponding to a throughput of approximately 20 images per second. This highlights the scalability and potential applicability of the model in high-throughput organoid imaging workflows.

### 3.3. Comparative Analysis

Figure 6 illustrates side-by-side comparisons of the original test images, ground-truth masks, and segmentation outputs generated by the proposed model. The enhanced U-Net-based algorithm demonstrated robust boundary detection even in challenging cases, such as organoids with irregular edges and variable morphologies. Quantitative results further confirmed these observations where the proposed method achieved a mean accuracy of 98.15%, a Dice similarity coefficient of 0.9719, and a Jaccard index of 0.9453 (Table 1), outperforming the comparative methods. Although a small proportion of organoid regions with highly complex textures were less clearly distinguished, overall segmentation quality remained consistently high across diverse morphologies.

These results demonstrate that integrating ROI refinement into the U-net architecture markedly improves segmentation performance for organoid imaging. Accurate delineation of boundaries is particularly important for downstream analyses, such as growth kinetics, morphological classification, and drug-response studies. By providing a fully automated and reproducible workflow, this approach significantly reduces the manual workload typically associated with organoid image analysis and minimises observer bias.

Compared with traditional algorithms (e.g., K-means [29], contour-based methods [30,31]), our model achieved substantially higher Dice and Jaccard scores, highlighting its suitability for complex biological datasets. Furthermore, when comparing within the proposed method, at the 95% confidence level, the means of the three metric groups were not statistically different (*p* = 0.6164), indicating consistent and reliable performance across all metrics. Importantly, this method can be readily scaled for high-throughput applications, enabling the systematic analysis of large organoid collections.

In recent years, several advanced deep-learning frameworks have been developed for organoid image segmentation. For instance, TransUNet have improved long-range feature integration but require extensive computational resources and are less suited for rapid analysis of large bright-field datasets [32]. In contrast, the enhanced U-Net presented in this study was intentionally designed for accessibility and efficiency while maintaining high segmentation accuracy enough for use on standard laboratory GPUs. Other organoid-specific U-Net models such OrgaExtractor, RDAU-Net, and OrganoID addresses different practical needs. For example, OrgaExtractor emphasises multi-scale shape extraction [33], RDAU-Net focuses on feature-attention refinement for brain tumour segmentation [34], and OrganoID is optimised for time-lapse tracking [35], while the proposed model in this study instead integrates a ROI refinement step that minimises background noise and enhances boundary delineation in static bright-field images, thus improving throughput for high-content screening.

The current model was trained and validated on images containing spatially separated organoids. Since the segmentation of overlapping organoids remains a recognised challenge in the field, future work will focus on extending the present approach to address bright-field images with overlapping organoids to further improve its applicability in high-density culture conditions.

## 4. Conclusions

In this work, we developed and validated an enhanced U-net-based deep-learning model for the segmentation of organoid images. Our results highlight the robustness of the proposed approach in handling organoids with diverse shapes and irregular boundaries. This model can be employed to analyse organoid-based disease modelling and high-throughput drug screening, advancing personalised medicine. We acknowledge that the current study was limited to bright-field images and a relatively constrained dataset. Future work will focus on extending the model to multimodal imaging platforms, including fluorescence microscopy, live-cell tracking, and, potentially, medical imaging formats such as MRIs and X-rays, which will further strengthen scalability and clinical relevance.

## Figures and Tables

**Table 1:** Performance comparison of segmentation methods compared with the proposed system.

Methods	Accuracy	Dice Similarity Coefficient	Jaccard Index
Hölder mean-based image [23]	0.9198 ± 0.001	0.9456 ± 0.001	0.8967 ± 0.002
K-means segmentation method [24]	0.776 ± 0.002	0.8739 ± 0.002	0.7761 ± 0.0011
Deep-learning semantic segmentation [25]	0.9198 ± 0.002	0.4776 ± 0.003	0.4137 ± 0.002
Proposal Model	0.9815 ± 0.007 *	0.9718 ± 0.007 *	0.94537 ± 0.012 *

Data represents Means ± Standard Deviations. * *p* < 0.05 via one-way ANOVA.

## Data Availability

All data needed to evaluate the conclusions in the paper are present in the paper and further inquiries can be directed to the corresponding author(s). The code of the developed model has been deposited to GitHub repository (https://github.com/Hamid-Jalab/segmentation-model-of-Organoid-Images/tree/main).

## References

[1] Shaker M.R., Kahtan A., Prasad R., Lee J.-H., Pietrogrande G., Leeson H.C., Sun W., Wolvetang E.J., Slonchak A. (2022). Neural Epidermal Growth Factor-Like Like Protein 2 Is Expressed in Human Oligodendroglial Cell Types. Front. Cell Dev. Biol..

[2] Shaker M.R., Aguado J., Chaggar H.K., Wolvetang E.J. (2021). Klotho inhibits neuronal senescence in human brain organoids. npj Aging Mech. Dis..

[3] Shaker M.R., Slonchak A., Al-mhanawi B., Morrison S.D., Sng J.D.J., Cooper-White J., Khromykh A.A., Wolvetang E.J. (2024). Choroid plexus defects in Down syndrome brain organoids enhance neurotropism of SARS-CoV-2. Sci. Adv..

[4] Lee J.-H., Shin H., Shaker M.R., Kim H.J., Park S.-H., Kim J.H., Lee N., Kang M., Cho S., Kwak T.H. (2022). Production of human spinal-cord organoids recapitulating neural-tube morphogenesis. Nat. Biomed. Eng..

[5] Lee J.-H., Shaker M.R., Park S.-H., Sun W. (2023). Transcriptional Signature of Valproic Acid-Induced Neural Tube Defects in Human Spinal Cord Organoids. Int. J. Stem Cells.

[6] Al-mhanawi B., Marti M.B., Morrison S.D., Gupta P., Alani M., Noakes P.G., Wolvetang E.J., Shaker M.R. (2023). Protocol for generating embedding-free brain organoids enriched with oligodendrocytes. STAR Protoc..

[7] Shaker M.R., Hunter Z.L., Wolvetang E.J. (2022). Robust and Highly Reproducible Generation of Cortical Brain Organoids for Modelling Brain Neuronal Senescence In Vitro. JoVE.

[8] Clevers H. (2016). Modeling Development and Disease with Organoids. Cell.

[9] Guo Z., Li X., Huang H., Guo N., Li Q. (2019). Deep Learning-Based Image Segmentation on Multimodal Medical Imaging. IEEE Trans. Radiat. Plasma Med. Sci..

[10] Vijay H., Bhupendra S. (2014). Segmentation of Microscopic Images: A Survey. Conference. Proceedings of the International Conference on Electronic Systems, Signal Processing and Computing Technologies.

[11] Liu Z., Wang J., Liu G., Zhang L. (2019). Discriminative low-rank preserving projection for dimensionality reduction. Appl. Soft Comput..

[12] Huang C., Ding H., Liu C. (2020). Segmentation of cell images based on improved deep learning approach. IEEE Access.

[13] Colin F., Cisneros M.T., Cervantes J., Martinez J., Debeir O. (2006). Detection of biological cells in phase-contrast microscopy images. Proceedings of the Fifth Mexican International Conference on Artificial Intelligent MICAI.

[14] Liu Z., Lai Z., Ou W., Zhang K., Zheng R. (2020). Structured optimal graph based sparse feature extraction for semi-supervised learning. Signal Process..

[15] Havaei M., Davy A., Warde-Farley D., Biard A., Courville A., Bengio Y., Pal C., Jodoin P.-M., Larochelle H. (2017). Brain tumor segmentation with deep neural networks. Med. Image Anal..

[16] Ronneberger O., Fischer P., Brox T. U-net: Convolutional networks for biomedical image segmentation. Proceedings of the Medical Image Computing and Computer-Assisted Intervention–MICCAI 2015: 18th International Conference.

[17] Jia X., Bartlett J., Zhang T., Lu W., Qiu Z., Duan J. U-net vs. transformer: Is u-net outdated in medical image registration?. Proceedings of the International Workshop on Machine Learning in Medical Imaging.

[18] Gu Z., Cheng J., Fu H., Zhou K., Hao H., Zhao Y., Zhang T., Gao S., Liu J. (2019). Ce-net: Context encoder network for 2d medical image segmentation. IEEE Trans. Med. Imaging.

[19] Yi J., Wu P., Jiang M., Huang Q., Hoeppner D.J., Metaxas D.N. (2019). Attentive neural cell instance segmentation. Med. Image Anal..

[20] Loh D.R., Yong W.X., Yapeter J., Subburaj K., Chandramohanadas R. (2021). A deep learning approach to the screening of malaria infection: Automated and rapid cell counting, object detection and instance segmentation using Mask R-CNN. Comput. Med. Imaging Graph..

[21] Dumevska B., Bosman A., McKernan R., Schmidt U., Peura T. (2016). Derivation of human embryonic stem cell line Genea022. Stem Cell Res..

[22] Hunter Z.L., Leeson H.C., Shaker M.R., Wolvetang E.J., Vadlamudi L. (2021). Generation of induced pluripotent stem cell lines from peripheral blood mononuclear cells of three drug resistant and three drug responsive epilepsy patients. Stem Cell Res..

[23] Miura Y., Li M.-Y., Revah O., Yoon S.-J., Narazaki G., Pașca S.P. (2022). Engineering brain assembloids to interrogate human neural circuits. Nat. Protoc..

[24] Agarap A.F. (2018). Deep learning using rectified linear units (relu). arXiv.

[25] Xu B., Wang N., Chen T., Li M. (2015). Empirical evaluation of rectified activations in convolutional network. arXiv.

[26] Eelbode T., Bertels J., Berman M., Vandermeulen D., Maes F., Bisschops R., Blaschko M.B. (2020). Optimization for medical image segmentation: Theory and practice when evaluating with dice score or jaccard index. IEEE Trans. Med. Imaging.

[27] Vania M., Mureja D., Lee D. (2019). Automatic spine segmentation from CT images using convolutional neural network via redundant generation of class labels. J. Comput. Des. Eng..

[28] Rad R.M., Saeedi P., Au J., Havelock J. (2020). Trophectoderm segmentation in human embryo images via inceptioned U-Net. Med. Image Anal..

[29] Asvadi A., Karami M., Baleghi Y. (2011). Efficient object tracking using optimized K-means segmentation and radial basis function neural networks. Int. J. Inf. Commun. Technol.

[30] Altulea A.H., Jalab H.A., Ibrahim R.W. (2020). Fractional Hölder mean-based image segmentation for mouse behavior analysis in conditional place preference test. Signal Image Video Process..

[31] Chen L.-C., Zhu Y., Papandreou G., Schroff F., Adam H. Encoder-decoder with atrous separable convolution for semantic image segmentation. Proceedings of the European Conference on Computer Vision (ECCV).

[32] Chen J., Lu Y., Yu Q., Luo X., Adeli E., Wang Y., Lu L., Yuille A.L., Zhou Y. (2021). TransUNet: Transformers Make Strong Encoders for Medical Image Segmentation. arxiv.

[33] Park T., Kim T.K., Han Y.D., Kim K.-A., Kim H., Kim H.S. (2023). Development of a deep learning based image processing tool for enhanced organoid analysis. Sci. Rep..

[34] Wang J., Yu Z., Luan Z., Ren J., Zhao Y., Yu G. (2022). RDAU-Net: Based on a Residual Convolutional Neural Network with DFP and CBAM for Brain Tumor Segmentation. Front. Oncol..

[35] Matthews J.M., Schuster B., Kashaf S.S., Liu P., Ben-Yishay R., Ishay-Ronen D., Izumchenko E., Shen L., Weber C.R., Bielski M. (2022). OrganoID: A versatile deep learning platform for tracking and analysis of single-organoid dynamics. PLOS Comput. Biol..

